# Skeletal muscle fibers produce B-cell stimulatory factors in chronic myositis

**DOI:** 10.3389/fimmu.2023.1177721

**Published:** 2023-09-05

**Authors:** Per-Ole Carstens, Luisa M. Müllar, Arne Wrede, Sabrina Zechel, Martin M. Wachowski, Almuth Brandis, Sabine Krause, Stephan Zierz, Jens Schmidt

**Affiliations:** ^1^ Department of Neurology, University Medical Center Göttingen, Göttingen, Germany; ^2^ Institute of Neuropathology, University Medical Center Göttingen, Göttingen, Germany; ^3^ Institute of Neuropathology, Saarland University Medical Center and Medical Faculty of Saarland University, Homburg, Germany; ^4^ Department of Trauma Surgery, Orthopaedics and Plastic Surgery, University Medical Center Göttingen, Göttingen, Germany; ^5^ Department of Pathology, Klinikum Region Hannover, Hannover, Germany; ^6^ Institute of Pathology and Neuropathology, Medical University Hannover, Hannover, Germany; ^7^ Friedrich-Baur-Institute, Department of Neurology, Ludwig-Maximilians-University of München, München, Germany; ^8^ Department of Neurology, University Hospital Halle/Saale, Halle, Germany; ^9^ Department of Neurology and Pain Treatment, Neuromuscular Center, Center for Translational Medicine, Immanuel Klinik Rüdersdorf, University Hospital of the Brandenburg Medical School Theodor Fontane, Rüdersdorf bei Berlin, Germany; ^10^ Faculty of Health Sciences Brandenburg, Brandenburg Medical School Theodor Fontane, Rüdersdorf bei, Berlin, Germany

**Keywords:** autoimmune diseases, neuromuscular disease, inflammatory muscle disease, myositis, B cells

## Abstract

**Introduction:**

We aimed to identify B-cell-mediated immunomechanisms in inclusion body myositis (IBM) and polymyositis (PM) as part of the complex pathophysiology.

**Materials and methods:**

Human primary myotube cultures were derived from orthopedic surgery. Diagnostic biopsy specimens from patients with IBM (n=9) and PM (n=9) were analyzed for markers of B cell activation (BAFF and APRIL) and for chemokines that control the recruitment of B cells (CXCL-12 and CXCL-13). Results were compared to biopsy specimens without myopathic changes (n=9) and hereditary muscular dystrophy (n=9).

**Results:**

The mRNA expression of BAFF, APRIL, and CXCL-13 was significantly higher in IBM and PM compared to controls. Patients with IBM displayed the highest number of double positive muscle fibers for BAFF and CXCL-12 (48%) compared to PM (25%), muscular dystrophy (3%), and non-myopathic controls (0%). *In vitro*, exposure of human myotubes to pro-inflammatory cytokines led to a significant upregulation of BAFF and CXCL-12, but APRIL and CXCL-13 remained unchanged.

**Conclusion:**

The results substantiate the hypothesis of an involvement of B cell-associated mechanisms in the pathophysiology of IBM and PM. Muscle fibers themselves seem to contribute to the recruitment of B cells and sustain inflammation.

## Introduction

The group of idiopathic inflammatory myopathies consists of dermatomyositis (DM), polymyositis (PM), inclusion body myositis (IBM), necrotizing myopathy (NM), and anti-synthetase syndrome (ASS). Over the last few years, there was growing evidence for the involvement of B cells in idiopathic inflammatory myopathies. Especially in DM and ASS, the role of B-cells in the pathophysiology is well known and several autoantibodies are well characterized (1–3). The histological picture in NM is scattered necrotic myofibers and T- or B cells may be found in focal spots, but there are no primary inflammatory lesions (3).

The pathophysiology of IBM is very complex and, in general, characterized by mechanisms of inflammation and β-amyloid associated degeneration. Recent evidence suggests that inflammation may be the primary event. IBM and PM are considered to be T cell-mediated diseases, but high frequencies of plasma cells were detected in muscle biopsy specimens (4), and infiltrating B-cells and plasma cells were found to be clonally expanded and had undergone affinity maturation locally within the muscle (5–7). Plasma cells derived from IBM muscles produce autoantibodies that are directed against antigens in muscle tissue (e.g., Desmin) (8), and the autoantibody directed against cytosolic 5’-nucleotidase 1A (cN1A) was identified in IBM (9, 10). In pure PM, usually, no myositis-associated antibodies are found. Apart from immune cells, muscle fibers are considered capable of immune cell recruitment and T-cell activation (11). The reason for analyzing IBM and PM in this study as the only two subtypes of myositis is their similar pathophysiology especially regarding CD8 cytotoxicity (3) and the limited knowledge of B-cell mechanisms in both disorders.

IBM and PM were compared to muscular dystrophy as disease control. We did not compare the expression levels with DM, NM, and ASS because B-cell mechanisms in these disorders have been characterized before and we did not aim to repeat these observations.

Another aim of this study was to examine the pathophysiological contribution of muscle cells in the context of myositis and not to analyze the impact of other immune cells.

The B-cell-activating factor of the tumor necrosis family (BAFF) and a proliferation-inducing ligand (APRIL) are crucial for B cell survival, maturation, activation, and differentiation (12, 13). Trafficking of human naive and memory B-cells is mainly orchestrated by CXC-chemokine ligands (CXCL) 12 and 13 (14).

We studied the distribution of these factors associated with B cells and the *in vitro* effect of an inflammatory environment to drive and sustain a humoral immune response in the skeletal muscle.

## Materials and methods

### Patients

The project was approved by the institutional review board (ethics committee) and patients signed informed consent. Diagnostic biopsies were used from the skeletal muscle of patients with inclusion body myositis (n=9), polymyositis (n=9), muscular dystrophy (n=9), and non-myopathic controls (n=9). Most biopsies were obtained from the Department of Neuropathology of the University Medical Center Göttingen. In order to achieve comparable group sizes in IBM and PM, a few additional biopsies were obtained from the Departments of Neuropathology or Neurology of the University Medical Centers of München (PM; n=3), Halle (IBM; n=2), and Hannover (IBM; n=1) (all in Germany).

The anti-cN1A antibody status was documented in one out of nine IBM patients and that was negative.

### Muscle biopsies

Frozen sections (5µm) of muscle biopsy specimens or cultured muscle cells were fixed with 4% paraformaldehyde at room temperature (for hematoxylin and eosin staining) or acetone at -20°C (all other stainings) for 10 min. Unspecific binding was reduced by 45 min incubation with 10% bovine serum albumin (BSA) and 100% goat serum (all from Jackson ImmunoResearch, West Grove, USA). The following anti-human antibodies were used: rat anti-BAFF (1:1000, Abcam, Cambridge, USA) and mouse anti-CXCL-12 (1:1000, R&D Systems, Minneapolis, USA). The primary antibodies were diluted in BSA and incubated for 1h at room temperature. Immunoreactivity was detected using Alexa-488 or Alexa-594-conjugated goat antibodies against mice or rats (1:600, all from Molecular Probes, Leiden, Netherlands). Nuclear counterstaining was performed with DAPI (Molecular Probes/Invitrogen, Carlsbad, USA) at 1:50,000 for 1 minute, followed by mounting in Fluoromount-G (Southern Biotech, Alabama, USA). Immunofluorescence microscopy and digital photography were performed on an Axiovert 200m microscope (Zeiss, Oberkochen, Germany) using a 20x and 40x objective, appropriate filters for green (488 nm), red (594 nm), and blue (350 nm) fluorescence, and a cooled CCD digital camera (Retiga 1300; QImaging) using the QCapture software (QImaging). For every biopsy, photomicrographs that covered a cross-section of each biopsy specimen were taken by an investigator who was blinded to the source of the specimen (LM). Scion Image software was used for grey-scale analysis.

### Extraction of mRNA and reverse transcription–polymerase chain reaction

Total RNA was extracted from cell culture using a commercial kit (RNeasy from Qiagen, Venlo, Netherlands), following the supplier’s instructions. Total RNA from the muscle biopsy specimen was homogenized in 500 µl TRIzol (life technologies, Carlsbad, USA) with a plastic tissue grinder and pestle (Kimble Chase, Vineland, USA). Precipitation of mRNA was improved by Ambion 9.520 (life technologies, Carlsbad, USA). RNA was eluted in 30 µl RNA-ase free water and stored at -80°C. Complementary DNA (cDNA) synthesis was performed with SuperScript II reverse transcriptase (Invitrogen), following the supplier’s instructions. The resulting cDNA was stored at –20°C. Quantitative (real-time) polymerase chain reaction was performed as previously described (15) on an SDS 7500 Sequence Detection System (Applied Biosystems, Foster City, USA) by 6-carboxyfluorescein (FAM-) labeled specific primer/probes: Glycerinaldehyd-3-Phosphat Dehydrogenase (GAPDH) Hs99999905_m1, *B-Cell-Activating Factor* (BAFF) Hs00198106_m1, *A Proliferation-Inducing Ligand* (APRIL) Hs00182565_m1, CXCL-12 Hs00171022_m1, and CXCL-13 Hs00757930_m1 (from Applied Biosystems, Foster City, USA). The resulting mRNA expression was quantified using the Δc(t) method in relation to the expression of GAPDH mRNA.

### Cell culture stimulation studies

As described previously (15, 16), satellite cells -muscle cell progenitors- from diagnostic biopsy specimens from patients without myopathic changes were grown according to the following protocol: The muscle piece was minced and washed in phosphate-buffered saline and trypsinized. The fragments were seeded to a 25 cm^2^ flask in Dulbecco’s modified Eagle’s medium with pyruvate, high glucose, and L-glutamine (Gibco Invitrogen, Carlsbad, USA), supplemented with 10% fetal calf serum (Cambrex Bioscience), penicillin, streptomycin (Gibco Invitrogen, Carlsbad, USA), and 0.5% chick embryo extract (Accurate). After 21 days, myotubes were labeled with neural cell adhesion molecules (anti-CD56, mouse clone Eric-1; Neomarkers/Labvision), followed by magnetic bead–labeled secondary antibodies and subsequently separated by magnets (Dynal/Invitrogen, Carlsbad, USA). For further experiments, myotubes were seeded either in 8-chamber slides (LabTek II; Nunc) or 24-well plates (Nunc), and at 80% confluence, fusion was induced by serum deprivation. Well-differentiated myotubes, as revealed by immunocytochemical staining for the muscle marker desmin, were either kept as unstimulated controls in X-Vivo 15 medium (Cambrex Bioscience) and exposed to the cytokines IFN-γ (300 units/ml), TNFα (10 ng/ml), and IL-1β (20 ng/ml) (all from Chemicon International Inc., Temecula, USA) in serum-free X-Vivo 15 medium. The duration of incubation was 12 to 48h. Scion Image software was used for grey-scale analysis.

### Statistical analysis

For statistical analysis (ANOVA, t-test, Kruskal-Wallis, and Pearson correlation), *P< 0.05, **P< 0.01, and ***P< 0.001 were used as significant values, and all significant outliers (Grubb’s test) were excluded prior to analysis. Statistical analyses were performed using the software GraphPad Prism 7 (San Diego, CA, USA).

## Results

### Upregulation of factors associated with B-cell activation and chemotaxis in IBM and PM

**Figure 1:**
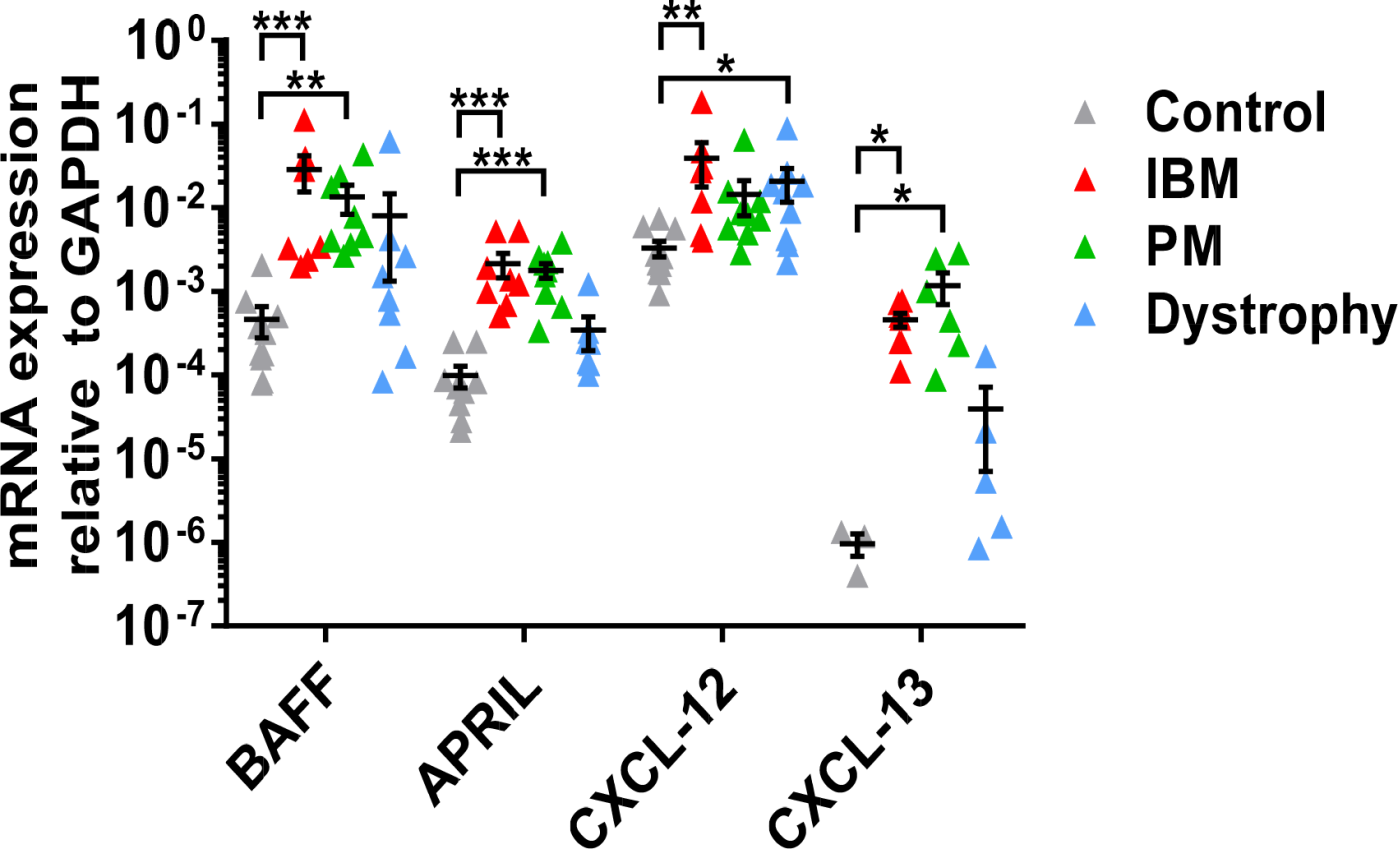
mRNA expression of BAFF, APRIL, CXCL-12, and CXCL-13 in muscle tissue specimens by quantitative real-time-PCR: Significant upregulation of BAFF, APRIL, and CXCL-13 in IBM and PM and of CXCL-12 in IBM compared to non-myopathic controls. Comparable results for all molecules in IBM and PM. Upregulation of CXCL-12 in muscular dystrophies compared to non-myopathic controls. Data shown as mean +SD. Statistics using multiple comparisons by Kruskal-Wallis testing, indicating significance by *P< 0.05; **P< 0.01; ***P< 0.001. IBM, inclusion body myositis; PM, polymyositis; Dystrophy, muscular dystrophies; BAFF, B-cell-activating factor of the tumor necrosis family; APRIL, A proliferation-inducing ligand, CXCL-12, CXC-chemokine ligand 12; CXCL-13, CXC-chemokine ligand 13.

We first studied the mRNA expression of factors associated with B-cell activation and chemotaxis in muscle specimens from patients with IBM, PM, muscular dystrophies, and non-myopathic controls. In IBM compared to non-myopathic controls, there was significant overexpression of BAFF (mean 0.028 ± 0.037, P=0.0004), APRIL (mean 0.0021 ± 0.0019, P=0.001), CXCL-12 (mean 0.039 ± 0.06, P=0.003), and CXCL-13 (mean 0.00046 ± 0.00024, P=0.04) (**Figure 1**). In PM compared to non-myopathic controls, we noted a significant overexpression of BAFF (mean 0.013 ± 0.014, P=0.002), APRIL (mean 0.0017 ± 0.001, P=0.0002), and CXCL-13 (mean 0.0011 ± 0.0012, P=0.04) but not for CXCL-12 (mean 0.0014 ± 0.0019, P=0.067). The expression of all four targets was comparable in PM and IBM and no differences were noted. In comparison to muscular dystrophies, the expression of APRIL and CXCL-13 in IBM and PM showed a tendency for an upregulation, which did not reach statistical significance. CXCL-12 was significantly upregulated in muscular dystrophies compared to non-myopathic controls (mean 0.021 ± 0.026, P=0.03).

Collectively, the B cell-associated factors BAFF and APRIL as well as the B-cell chemoattractants CXCL-12 and 13 were upregulated in muscle specimens from patients with IBM and PM compared to non-myopathic controls. Only CXCL-12 was upregulated in dystrophic muscle compared to non-myopathic controls.

### Upregulation of BAFF and CXCL-12 on protein levels in IBM and PM

**Figure 2:**
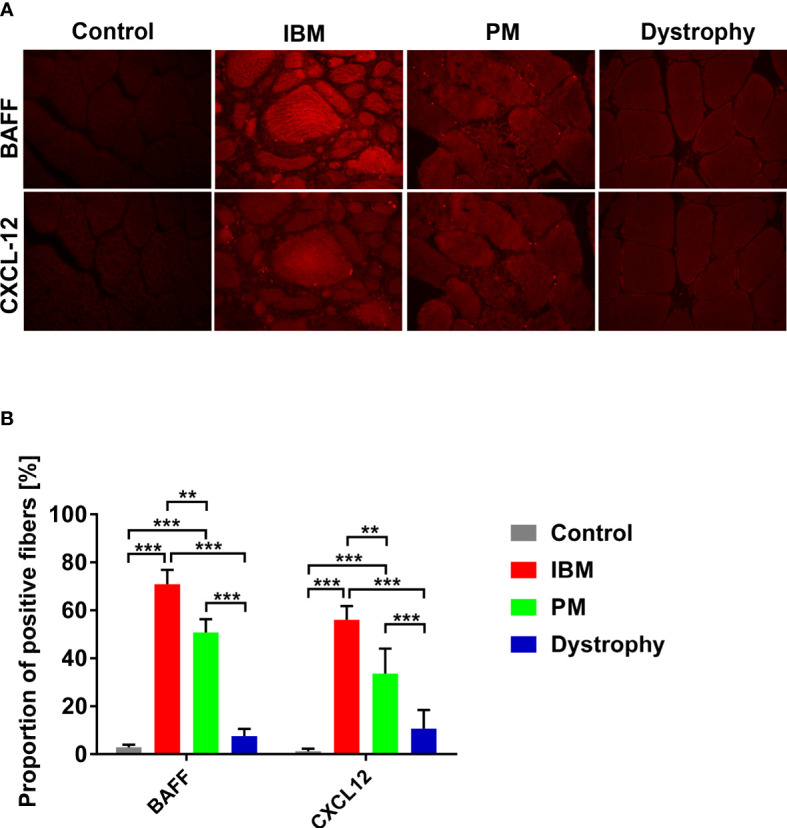
Analysis of BAFF and CXCL-12 expression on protein levels in IBM and PM by immunohistochemistry: **(A)** Exemplary findings of muscle tissue specimen of IBM, PM, dystrophies, and muscle control tissue in BAFF and CXCL-12 immunostainings (microphotographs acquired using a 20x objective). **(B)** Significantly increased numbers of BAFF and CXCL-12 positive muscle fibers in IBM and PM compared with non-myopathic controls and muscular dystrophies. For the analysis, 78 to 233 myofibers were counted in each specimen. Similar results are found in the automated grey scale analysis (data not shown). Data shown as mean +SD. Statistics using ANOVA with Tukey’s multiple comparison test, indicating significance by **P< 0.01; ***P< 0.001. IBM, inclusion body myositis; PM, polymyositis; Dystrophy, muscular dystrophies; BAFF, B-cell-activating factor of the tumor necrosis family; CXCL-12, CXC-chemokine ligand 12.

At the protein level, overexpression of BAFF and CXCL-12 in muscle specimens was confirmed by immunohistochemistry (exemplary findings are shown in **Figure 2A**). Manual counting of the number of fibers in each specimen (range from 78 to 233) in a blinded fashion revealed a significantly higher number of muscle fibers positive for BAFF and CXCL-12 in IBM (BAFF 70%, CXCL-12 56%, and double positive 48%) compared to PM (BAFF 50%, CXCL-12 33%, and double positive 25%). Both forms of myositis displayed a significantly increased number of positive fibers in comparison to muscular dystrophies (BAFF 7%, CXCL-12 10%, and double positive 3%) and non-myopathic controls (BAFF 2.9%, CXCL-12 1.2%, and double positive 0%) (**Figure 2B**). Similar results were observed by automated grey scale analysis (data not shown).

Our data demonstrate that major sources of BAFF and CXCL-12 are muscle fibers themselves. Taken together, these data suggest that the local inflammatory milieu generated by muscle fibers could directly contribute to the recruitment and activation of B cells in IBM and PM.

### Strong induction of BAFF by IFN-γ and of CXCL-12 by IL-1β on mRNA levels in human myotubes

**Figure 3:**
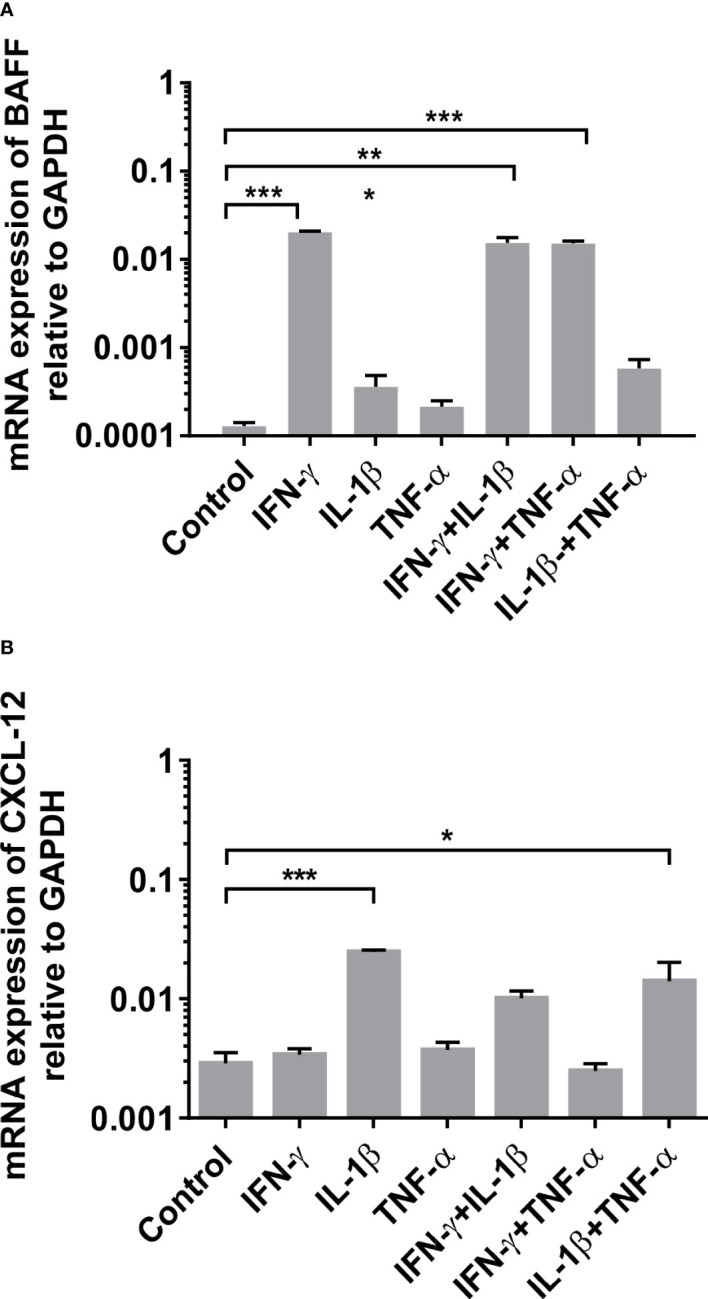
mRNA expression of BAFF and CXCL-12 in human myotubes under pro-inflammatory conditions by quantitative (real-time)-PCR after 24 hours of stimulation: **(A)** Significant induction of BAFF mRNA expression upon IFN-γ (alone or in combination) and of **(B)** CXCL-12 upon IL-1β (alone or in combination) in human primary myotube cultures. The results are averages of two independent experiments and the myotubes were obtained from non-myopathic donors. BAFF, B-cell-activating factor of the tumor necrosis family; CXCL-12, CXC-chemokine ligand 12. IFN-γ, Interferon γ; IL-1β, Interleukin 1β; TNFα, Tumor necrosis factor α. Data shown as mean +SD. Statistics by ANOVA with Tukey's multiple comparison test, indicating significance by *P< 0.05; **P< 0.01; ***P< 0.001.

Based on our biopsy findings, we assessed the effect of an inflammatory environment on human skeletal muscle *in vitro*. Human primary myotube cultures were incubated for 24 hours in the presence of the cytokines IFN-γ, IL-1β, and TNF-α alone or in combination, and the induction of BAFF, APRIL, CXCL-12, and CXCL-13 mRNA in muscle cells was measured by qPCR. We noted a strong and significant induction of BAFF by IFN-γ alone or in combination with any other proinflammatory cytokine (**Figure 3A** **;** IFN-γ: mean 0.02 ± 0.00067, P<0.001; IFN-γ + IL-1β: mean 0.015 ± 0.00096, P<0.001; IFN-γ + TNFα: mean 0.015 ± 0.00015, P<0.001). For CXCL-12, similar results were observed upon IL-1β alone or in combination with TNF-α (**Figure 3B** **;** IL-1β: mean 0.024 ± 0.00088, P=0.005; IL-1β + TNF-α: mean 0.014 ± 0.0062, P=0.026). The combination of IL-1β and IFN-γ did upregulate the expression of CXCL-12, but this did not reach statistical significance compared to controls (**Figure 3B** **;** IL-1β + IFN-γ: mean 0.01 ± 0.0015, P=0.17). After 12, 36, and 48 hours of stimulation, similar results were found (data not shown). The assessment for APRIL and CXCL-13 revealed no changes of mRNA levels under proinflammatory conditions (data not shown). The results are averages of two independent experiments and the myotubes were obtained from healthy donors.

### Increased protein production of BAFF and CXCL-12 protein under proinflammatory conditions in human myotubes

**Figure 4:**
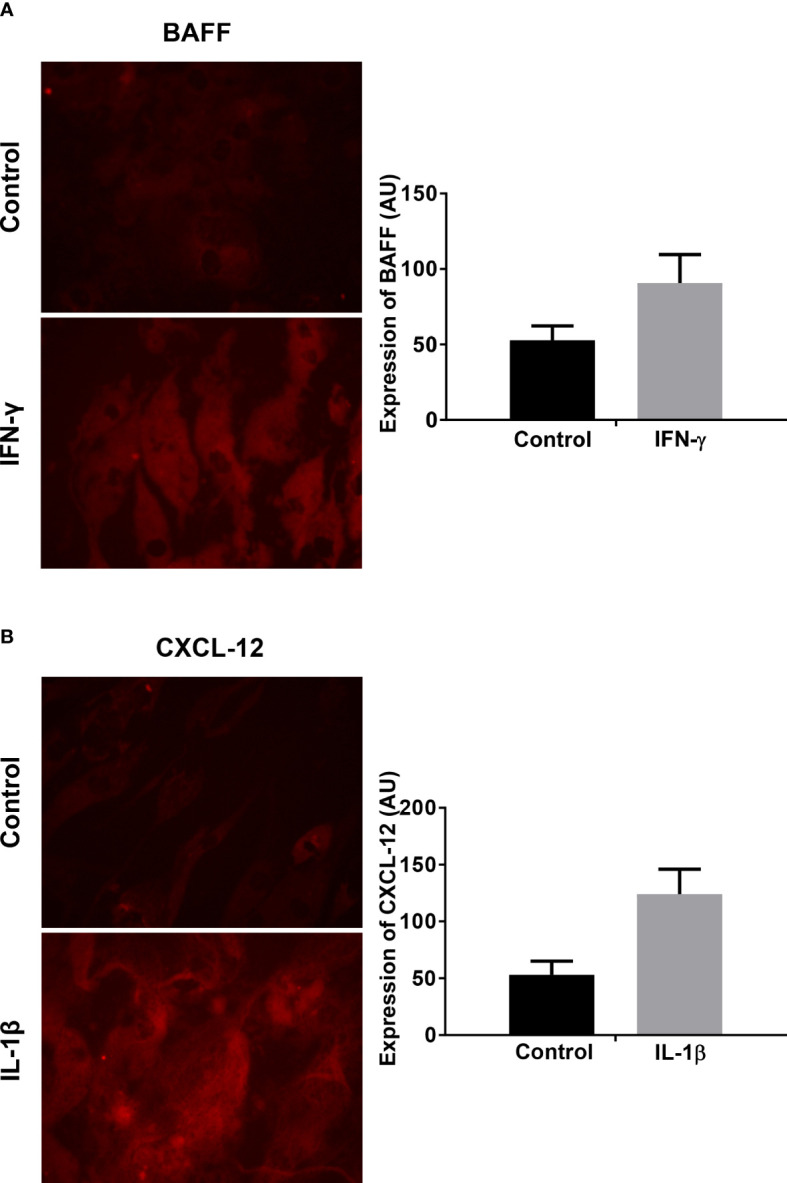
Analysis of BAFF and CXCL-12 expression on protein levels in human myotubes under pro-inflammatory conditions by immunocytochemistry: **(A)** Exemplary finding of BAFF expression in human myotubes after 48 hours exposure to IFN-γ compared to controls (microphotographs acquired using a 20x objective). Greyscale analysis revealed a tendency of an increased protein production, which did not reach statistical significance (t-test). **(B)** Exemplary finding of CXCL-12 expression in human myotubes after 48 hours exposure to IL-1β compared to controls (microphotographs acquired using a 20x objective). Greyscale analysis revealed a tendency of an increased protein production, which did not reach statistical significance (t-test). The myotubes were obtained from non-myopathic donors. The results are shown as mean +SD from two independent experiments. BAFF, B-cell-activating factor of the tumor necrosis family; CXCL-12, CXC-chemokine ligand 12. IFN-γ, Interferon γ; IL-1β, Interleukin 1β.

For the analysis of the protein expression, myotubes were incubated for 12, 24, or 48 hours with IFN-γ and IL-1β and afterwards stained with anti-BAFF and anti-CXCL-12 antibodies. Exemplary findings of the immunocytochemical staining for BAFF and CXCL-12 are shown in **Figure 4**. The staining revealed an upregulation of both factors upon exposure to IFN-γ for BAFF or to IL-1β for CXCL-12, respectively. By grey scale analysis, the upregulation of BAFF upon exposure to IFN-γ (**Figure 4A**; mean 52 ± 6 vs. 90 ± 13, P=0.12) and the induction of CXCL-12 upon IL-1β (**Figure 4B** **;** mean 53 ± 8 vs. 124 ± 15, P=0.057) did not reach statistical significance compared to controls. The results are averages of two independent experiments.

Collectively, these data suggest that non-diseased human myotubes can express factors involved in B cell activation and cell recruitment upon exposure to proinflammatory cytokines.

## Discussion

The underlying pathomechanisms of myositis are complex and only partially understood. IBM and PM are thought to be T cell-mediated disorders as infiltrates of T cells can be noted in muscle biopsies. Over the last years, B-cell mechanisms in myositis have drawn more attention and recent results support their contribution to various forms of myositis including PM and IBM (4–7). The role of B-cells is well known in DM and ASS and several autoantibodies were well characterized in these subtypes of myositis (1–3).

In this study, we demonstrate that the B-cell activating factors BAFF and APRIL as well as the chemokines CXCL-12 and CXCL-13 are upregulated on the mRNA level in muscle specimens from IBM and PM compared to controls. An elevated protein expression of BAFF and CXCL-12 in skeletal muscle biopsies was consistent with the upregulated mRNA findings in IBM and PM. Human primary myotubes derived from individuals without a neuromuscular disease produced BAFF and CXCL-12 mRNA and protein upon exposure to proinflammatory cytokines.

The immunohistochemical analysis identified muscle fibers themselves as sources of BAFF and CXCL-12 in PM and IBM. We were unable to detect these markers on or around inflammatory cells, which is likely explained by an insufficient sensitivity of the antibodies used.

Elevated serum levels for BAFF were reported for autoimmune muscular disorders like DM, PM (17–20), and Myasthenia gravis (21) but not for IBM (22). APRIL was identified in unspecified inflammatory muscle disorders only in one study (17). A significant upregulation of BAFF mRNA was reported in muscle specimens of IBM, PM, and DM (6, 23), and CXCL-13 mRNA was significantly upregulated in juvenile DM (24). A recent study observed elevated serum levels of CXCL-13 and other pro-inflammatory cytokines and chemokines in DM and ASS compared to healthy controls (25), yet CXCL-12 was not analyzed.

The expression of APRIL and CXCL-12 mRNA in muscle tissues as well as the expression of BAFF and CXCL-12 mRNA in human myotubes had not been analyzed before. We were able to confirm the finding of increased levels of BAFF mRNA in IBM and PM and could extend the findings to the expression of its ligand APRIL and the chemokines CXCL-12 and 13 in muscle tissue specimens of patients with IBM and PM.

On the protein level, few studies analyzed the expression of factors involved in B-cell activation in myositis. BAFF expression was detected by immunohistochemistry in perifascicular muscle cells in DM while no expression was found in blood vessels and normal controls (23). The expression of BAFF or APRIL in muscle cells in IBM and PM had not been reported so far, but the expression of their receptors had been analyzed by immunohistochemistry: BAFF-R, BCMA, and TACI were detected on mononuclear cells in the muscle tissue of patients with myositis (26). The highest rate of these receptors was found in IBM in five out of six analyzed patients, followed by PM (five out of 11) and DM (two out of 6). CXCL-12 was observed in macrophages (CD68+) and T cells (CD4+) invading muscle fibers (27) as well as in muscle fibers in IBM and PM (28). The protein amount of CXCL-12 evidenced by western blot was significantly increased in IBM and PM compared to healthy controls with a tendency for a higher expression in IBM (27); CXCL-13 localized to lymphoid follicle-like structures in juvenile DM and was absent in healthy muscles (24). In DM, CXCL-12 was shown to be strongly upregulated in affected blood vessels.

In DM, muscle fibers were positive for BAFF (23), and in IBM and PM, they stained positive for CXCL-12 (28). Our present data clearly demonstrate a substantial protein expression of BAFF in IBM and PM, which had not been reported before. In the present study, we demonstrated that muscle cells derived from non-myopathic subjects *in vitro* start to produce BAFF and CXCL-12 under proinflammatory conditions. This would be a substantial prerequisite for the induction of skeletal muscle inflammation *in vivo*. Muscle cells are known to be active participants rather than passive targets of an immune response (11) and, thus, exert a plethora of immune mediators (29). *In vivo* muscle cells have been shown to be capable of secreting proinflammatory cytokines such as IL-1α, IL-1β, TNFα, IL-6, IL-2, and IFN-γ and chemokines such as CXCL-12, and C-C motif ligand 2 (CCL2).

BAFF is known to be induced by the stimulation of IFN-α and IFN-γ (30) in salivary gland epithelial cells and in intestinal epithelial cells (31). The transcriptional up-regulation of the gene CXCL-12 can be induced in human and rat cell lines by inflammatory stimuli such as interleukin-1 and interleukin-6 (32). Our cell culture data are well in line with these findings as we were able to show a significant upregulation of BAFF and CXCL-12 mRNA in myotubes from non-myopathic individuals upon exposure to IFN-γ and IL-1β. Satellite-cell-derived myoblasts in rats express CXCL-12 protein levels with the highest amount when mature myotubes are formed (33). The staining characteristics are similar to our study with human myotubes. The finding of CXCL-12 expressed by normal muscle cells has not been reported for humans so far.

Taken together, our data demonstrate unique patterns of B cell regulatory factors in IBM and PM. Besides the known roles of T cells and macrophages in both diseases, a crucial role in the composition and regulation of muscle inflammation appears to be controlled by an interplay of muscle fibers and B cells through B cell signaling factors.

The regulation of BAFF and APRIL in autoimmune disorders of tissues other than skeletal muscle has been shown by different groups: In Lupus with the affection of kidneys and the central nervous system, serum BAFF has been shown to be elevated and serum APRIL was decreased (34, 35). The analysis of cerebrospinal fluid (CSF) from patients with inflammatory brain disorders like multiple sclerosis and Lyme neuroborreliosis revealed significantly increased levels of BAFF, CXCL-12, and CXCL-13 compared to patients with non-inflammatory neurological diseases (36), and BAFF protein levels were upregulated in patients with neuro-Behçet’s disease (37). In other B-cell-mediated disorders like rheumatoid arthritis, the levels of APRIL and BAFF were found to be elevated in serum and synovial fluid and both levels correlated with disease activity (38–40). Tabalumab, an anti-BAFF monoclonal antibody, showed efficacy and safety in rheumatoid arthritis in phase two studies but did not meet the primary or secondary outcomes in a phase three study (41), and Belimumab, a biologic BAFF inhibitor, has been the first biologic agent licensed for Lupus therapy (35).

Our findings on muscle specimens from patients with myositis are comparable with inflammatory central nervous system disorders in that increased levels of BAFF, CXCL-12, and CXCL-13 are observed. By contrast, APRIL mRNA was elevated in the skeletal muscle of IBM and PM but did not seem to be operative in inflammatory central nervous system disorders.

An explanation for increased levels of CXCL-12 in myositis as well as in muscular dystrophies could be its known role during muscle regeneration: Research has shown that injecting CXCL-12 into a damaged muscle led to an increase of muscle weight and improved muscle histology with a decreased level of fibrosis (33). The upregulation of CXCL-12 may be an attempt of the muscle fibers to improve muscular regeneration. Moreover, upregulation of CXCL-12 and its receptor CXCR4 was noted in the gastrocnemius muscle of rats after repeated periods of daily running performance compared to non-running controls (42). This can be interpreted as the involvement of CXCL-12 in adaption to muscle exercise. These findings reflect the pleiotropic role of chemokines like CXCL-12 in different pathomechanisms of the muscle and it underscores its relevance to distinct functions in the muscle. Within the inflammatory milieu of myositis, chemokines can activate unique immunomodulatory pathways including B-cell activation and support muscle regeneration with CXCL-12.

Collectively, in inflammatory myopathies, we demonstrate the muscular presence of molecular factors that are crucial for B cell survival, maturation, activation, and differentiation (BAFF) (12, 13) as well as for B-cell chemotaxis (14) (CXCL-12 and 13). These factors together provide a prerequisite for sustained B-cell involvement in the pathogenesis of IBM and PM.

B-cell mechanisms and autoantibody production seem to play an important role in specific forms of myositis like IBM and PM and are thought to be less important in unspecific inflammatory settings like in muscular dystrophies. Our data emphasize the relevance of specific muscle inflammation in IBM and PM. This is of particular relevance in view of autoantibodies like cN1A and supports the rationale for therapeutic efforts to modulate the humoral (auto-) immunity by drugs such as immunoglobulins or B cell depletion by rituximab.

## Limitations

The main limitation of our study is the relatively small number of patients, which is due to the rare nature of the disease subsets studied. Although the number of muscle biopsies was clearly sufficient for a meaningful statistical analysis, the data cannot be generalized without confirmatory studies.

Our data provide novel insight into the complex pathophysiology of myositis and the role of B cells. Beyond the techniques used in the present study, it will be of interest in the future to design complementary approaches including: i) further analysis of mRNA regulation by an epigenetic approach, ii) secreted protein levels of cytokines and chemokines in muscle cell culture by ELISA and Western blot, and iii) intracellular metabolism including (immune)-proteasome by a metabolomic approach.

## Data availability statement

The raw data supporting the conclusions of this article will be made available by the authors, without undue reservation.

## Ethics statement

The studies involving human participants were reviewed and approved by the University Medical Center Göttingen, Germany. The patients/participants provided their written informed consent to participate in this study.

## Author contributions

P-OC performed parts of the experiments, analyzed the data, and wrote the article. LM performed most of the experiments. MW was responsible for the surgical preparation of skeletal muscle for expansion of progenitors (satellite cells). AW, AB, SK, SZe and SZi contributed muscle biopsy specimens and revised the manuscript for intellectual content. SZe contributed clinal and pathological data of patients and revised the manuscript. JS conceived the idea, conceptualized and supervised all experiments, and revised the manuscript. All authors contributed to the article and approved the submitted version.
